# Wound Gel with Antimicrobial Effects Based on Polyvinyl Alcohol and Functional Aryloxycyclotriphosphazene

**DOI:** 10.3390/polym15132831

**Published:** 2023-06-27

**Authors:** Pavel Yudaev, Irina Butorova, Vladimir Chuev, Vera Posokhova, Bogdan Klyukin, Evgeniy Chistyakov

**Affiliations:** 1 Department of Chemical Technology of Plastics, Mendeleev University of Chemical Technology of Russia, Miusskaya Sq., 9, 125047 Moscow, Russia; 2 Belgorod National Research University, Pobedy Street, 85, 308015 Belgorod, Russia; 3 Trade House VladMiVa, 308015 Belgorod, Russia

**Keywords:** wound dressing, silver, phosphazene, hydrogel, aerogel

## Abstract

A silver-containing gel based on polyvinyl alcohol and aryloxycyclotriphosphazene containing *β*-carboxyethenylphenoxy and *p*-formylphenoxy groups has been developed. Phosphazene was synthesized via the Doebner reaction from hexakis[(4-formyl)phenoxy]cyclotriphosphazene and malonic acid and characterized by ^1^H, ^13^C, and ^31^P NMR spectroscopy and MALDI-TOF mass spectrometry. The study of the gel using scanning electron microscopy showed that the gel contains open pores and can absorb wound exudate. The maximum water absorption capacity of the gel was 272%, which was reached after 80 min of testing. The antimicrobial activity of the obtained silver-containing gel was evaluated using the diffusion method. The gel was found to inhibit the growth of the main microorganisms in contact with the skin: the bacteria *S. aureus*, *P. aeruginosa*, *E. coli*, *B. subtilis*, *S. epidermidis*, and *C. stationis* and the fungus *C. albicans*. The study of the wound-healing effect of the gel *in vivo* showed a decrease in the wound area of the rabbit hind limb by 91.43% (*p* < 0.05) on the 10th day of observation and a decrease in the content of C-reactive protein in the rabbit blood serum by 1.3 times (*p* < 0.05).

## 1. Introduction

The problem of healing wounds of various etiologies (leg ulcers, bedsores, diabetic wounds, traumatic wounds, surgical wounds, cancer-related wounds, etc.) is relevant for specialists from various fields of knowledge, including medicine, pharmacology, materials science, etc. Chronic diabetic wounds, which are a complication of diabetes mellitus, are difficult to treat and have a long healing time [1]. In diabetes, more reactive oxygen species are produced in a hyperglycemic environment, which increases inflammation. Clinicians, drug delivery scientists, and patients are in a constant struggle with non-healing chronic wounds [2].

Traditional wound dressings (gauze dressings) stick to the wound, injure the granulation tissue formed during healing, and cannot provide sufficient wound drainage, since they do not have the necessary sorption capacity for wound exudate [3]. The macroporous structure of cotton gauze provides an ideal environment for the growth of pathogenic microorganisms on its surface, causing cross-wound infection in the wound area [4].

The most promising alternative to the above dressings is dressings based on polymer gels [5]. This is due to their ability to absorb a large amount of water or biological fluids [6,7], pH sensitivity [8], temperature [9], ease of application to the wound surface, and their ability to maintain their three-dimensional structure without dissolution. Furthermore, they create a humid environment, allow oxygen diffusion into the wound, adsorb wound exudate, and act as a protective barrier against microorganisms [10]. Hydrogels create the desired microenvironment for the proliferation and differentiation of dermal fibroblasts, promoting angiogenesis, effective tissue repair, and skin regeneration [11]. In addition, hydrogels facilitate the removal of the wound dressing without secondary damage and the risk of infection [12].

Matrix transdermal systems based on polymer gels provide transdermal drug delivery, in particular delivery of antibiotics, through the epidermal layer of the skin into the systemic circulation to prevent bacterial infection after skin injury. The advantage of this delivery method is the reduction in side effects due to the absence of contact with the gastrointestinal tract, improved bioavailability, and maximized therapeutic effect of the drug at its minimum concentration [13].

Covalently cross-linked polymers obtained through the Diels–Alder reaction [14], non-covalently cross-linked non-toxic cryogels of chitosan and polyvinyl alcohol [15], gum arabic cryogels [16], nanofibers based on biodegradable poly-ε-caprolactone, and poly-L-lactide obtained via centrifugal spinning or electrospinning [17,18,19,20,21] have been used as gels to prepare wound dressings. Hydrogels based on carboxymethyl cellulose [22] and its combination with carbomer 940 [23], carboxymethyl chitosan [24,25], and chitosan with bacterial cellulose [26] are used to treat chronic wounds and burns.

Of considerable interest are wound dressings based on polyvinyl alcohol (PVA), which has biocompatibility, transparency, high water uptake, non-toxicity, and the ability to maintain a humid environment [27]. In addition, wound dressings based on PVA demonstrate high mechanical strength, which supports cell adhesion [28]; therefore, PVA is also used as an additive to gels based on other polymers (carboxymethyl cellulose, chitosan) to increase their stability [25]. However, PVA is devoid of antimicrobial activity, which can lead to bacterial colonization of PVA-based dressings. Therefore, antimicrobial additives must be added to PVA-based wound dressings.

The currently used topical antibiotics can cause allergic reactions and induce drug resistance of the main microorganisms that colonize the wound, such as *S. aureus* or *P. aeruginosa* [29]. Antiseptics for wound healing such as hydrogen peroxide, povidone-iodine, silver sulfadiazine, or chlorhexidine, are cytotoxic with respect to all major groups of cells involved in the wound process [30]. Therefore, a large number of studies are currently devoted to the use of nanoparticles as additives to wound-healing materials. These additives include nanoparticles of silver, gold, copper, zinc oxides, titanium dioxide, hematite, and copper(I) iodide, quantum dots, etc. [31,32,33,34,35,36,37,38,39]. Melamine nanoparticles introduced into the chitosan matrix accelerate the healing of infected diabetic wounds due to the rapid absorption of anti-inflammatory cytokines and reactive oxygen species, the high concentration of which in diabetic patients prevents and impairs wound healing [40]. Zinc oxide nanoparticles bind to thiol groups of bacterial enzymes and inhibit amino acid metabolism in bacteria. Zinc oxide nanoparticles have a greater inhibitory activity against Gram-positive bacteria and are less effective against Gram-negative ones [41]. Silver nanoparticles, unlike other nanoparticles, are available and cheap; have antimicrobial activity against a wide range of Gram-positive and Gram-negative bacteria in wounds, which is due to their large surface area-to-volume ratio [42]; and the ability to bind to bacterial cell membrane proteins and disrupt their function, thus causing the death of bacterial cells [43]. The use of polyvinyl alcohol with the addition of silver nanoparticles attached to a collagen matrix affords a skin substitute material that protects the wound from infections and from dehydration with minimal inflammatory reactions. This material can be used in patients with burn wounds [44].

Wound healing is a complex process consisting of four stages: hemostasis or coagulation, inflammation, cell proliferation, and remodeling of the newly formed extracellular matrix [45,46]. The first stage is accompanied by the formation of a fibrin platelet clot that stops bleeding. In the second stage of the process, infiltration of phagocytes (neutrophils and macrophages) occurs. Neutrophils migrate to the wound site and phagocytize foreign bodies and bacteria, thus destroying them and releasing proteolytic enzymes and reactive oxygen species. The presence of neutrophils is limited to the early stages of healing, while macrophages persist throughout the process. Along with phagocytes, silver ions also generate reactive oxygen species, causing damage to the bacterial DNA [47]. In the case of chronic wounds (venous, diabetic, bedsores), the healing process stops at the stage of inflammation, which is due to autoimmune insufficiency [48]. At the proliferation stage, wound closure is initiated. The wound site is filled with granulation tissue, and epithelialization occurs from the edges of the wound. At the stage of regeneration, macrophages die due to apoptosis, the normal structure of the dermis is restored, and the final formation of scar tissue occurs.

Due to the instability of their aqueous colloid, silver nanoparticles can aggregate in the gel matrix, which may lead to the loss of their antimicrobial activity and cause toxic effects on epithelial cells and fibroblasts and side effects for patients [49,50,51]. Potential side effects of silver nanoparticles include argyria, which manifests as a blue-gray discoloration of the skin [52], argyrosis (silver deposition in the eyes), hypersensitivity, and gastrointestinal disorders. Therefore, it is very important to distribute the silver evenly in the polymer gel matrix.

In the present study, we decided to use a new compound, aryloxycyclotriphosphazene containing *p*-formylphenoxy and *β*-carboxyethenylphenoxy (CFPP) groups, to prevent silver aggregation. Due to the presence of several aldehyde groups, CFPP cross-linked PVA macromolecules to form a gel, and β-carboxyethenylphenoxy groups allowed silver to be bound in the form of cinnamates, which provides an antimicrobial effect. Owing to the method used to combine CFPP and PVA solutions, phosphazene, and hence silver, were distributed throughout the entire volume of the resulting gel and were not washed out into the tissues surrounding the wound.

## 2. Materials and Methods

### 2.1. Materials

Malonic acid, tetrahydrofuran (THF), pyridine, piperidine, the deuterated solvent (DMSO-d_6_) for NMR analysis, polyvinyl alcohol (PVA), hydrochloric acid, 4-hydroxybenzaldehyde, ethanol, sodium, chloroform, potassium hydroxide, and silver nitrate were products of Sigma Aldrich (Saint Louis, MO, USA). Collagen types I and II were purchased from Proteintech Group Inc. (Chicago, IL, USA). Microbial strains were purchased from BioVitrum (Saint Petersburg, Russia). The antibiotic Nitox was purchased from NITA-FARM (Saratov, Russia).

### 2.2. Methods

^1^H, ^13^C, and ^31^P NMR spectra were recorded on an Agilent/Varian Inova 400 spectrometer (Agilent Technologies, Santa Clara, CA, USA) at 400.02 MHz, 100.06 MHz, and 161.94 MHz, respectively.

IR spectra were measured on a Nicolet 380 spectrometer (Thermo Fisher Scientific, Waltham, MA, USA) in the spectral range of 4000–500 cm^−1^ with a wavenumber accuracy of 0.01 cm^−1^.

The mass spectrum was recorded on a Microflex LRF mass spectrometer (Bruker Daltonic GmbH, Leipzig, Germany). 2,5-Dihydroxybenzoic acid was used as a matrix.

The silver content of the gel was determined on an X-Max SDD Inca Energy-Dispersive Spectrometer for electron probe microanalysis (Oxford Instruments, Abingdon, UK).

The content of the soluble fraction in the aerogel was determined in a Soxhlet apparatus. For this, 1 g of the gel was placed in a filter paper envelope, and extraction with tetrahydrofuran was carried out. The sample was dried, and the soluble fraction was determined from the weight difference.

The images of aerogel samples were obtained on a JEOL 1610LV scanning electron microscope (JEOL, Akishima, Japan) with an accelerating voltage of 0.5 to 30 kV and a maximum magnification of up to ×300,000.

The antimicrobial activity of the gels was evaluated using the diffusion method in accordance with MUK 4.2.1890-04 for determining the sensitivity of microorganisms to antibacterial drugs. The following strains of microorganisms were used as test organisms: *Escherichia coli* ATCC 25922, *Staphylococcus aureus* FDA 209P, *Pseudomonas aeruginosa* B-8243, *Candida albicans* Y-3108, *Bacillus subtilis* B-13183, *Staphylococcus epidermidis* B-6304, and *Corynebacterium stationis* B-10645. The diffusion method using wells (holes) was chosen. A sample (10 mg) was added to each well, and sterile distilled water (100 µL) was added.

The water absorption capacity (%WAC) of the silver-containing gel was determined by immersing a 1 cm^3^ sample into distilled water until the gel reached a constant weight.

The average performance characteristics for various samples were compared using two-way ANOVA followed by the Tukey’s range test for multiple comparisons.

### 2.3. Synthesis of Hexakis(4-formylphenoxy)cyclotriphosphazene (FPP)

Hexakis(4-formylphenoxy)cyclotriphosphazene was synthesized as described in a previous study [53].

4-Hydroxybenzaldehyde (7.32 g, 0.06 mol) was dissolved in ethanol (30 mL) in a three-necked flask equipped with a stirrer and a reflux condenser. After complete dissolution of 4-hydroxybenzaldehyde, an ethanol solution of sodium ethoxide, which was obtained via the dissolution of sodium (1.15 g, 0.05 mol) in ethanol (20 mL), was loaded into the flask. The reaction time was 10 min. Then, ethanol was distilled off on a rotary evaporator in vacuo. The residue was dried in vacuo to a constant weight. The yield of the product was quantitative.

4-Hydroxybenzaldehyde phenolate (8.64 g, 0.06 mol) was loaded into a three-necked flask equipped with a stirrer and a reflux condenser, and tetrahydrofuran (40 mL) was added. A solution of hexachlorocyclotriphosphazene (2.61 g, 0.0075 mol) in tetrahydrofuran (20 mL) was added to the dispersion formed on stirring. The reaction time in refluxing solvent was 9 h. When the reaction was complete, the reaction mixture was filtered, and the mother liquor was evaporated on a rotary evaporator. The product was recrystallized from an ethanol–chloroform mixture. Yield: 4.52 g (70%).

### 2.4. Synthesis of β-carboxyethenylphenoxy-p-formylphenoxycyclotriphosphazene (CFPP)

A 50 mL round-bottom flask equipped with a reflux condenser, a calcium chloride tube, and a magnetic stirrer was charged with FPP (1 g, 1.161 mmol) and dissolved in 20 mL of pyridine. Then, malonic acid (0.3626 g, 3.484 mmol) and 1 drop of piperidine were added into the flask. The reaction mixture was stirred at the solvent boiling point (115.6 °C) until the evolution of carbon dioxide ceased. The reaction mixture was precipitated with an aqueous solution of hydrochloric acid (50 mL of 36 wt. % hydrochloric acid in 250 mL of water). The resulting solid was separated from the liquid phase via centrifugation and washed several times with distilled water. The solid was dried in vacuo at 100 °C to a constant weight. The product was a white powder. Yield: 1.06 g (92.2%).

### 2.5. Preparation of a Gel Based on PVA and CFPP

A solution of PVA (5.2 g) in distilled water (26.3 mL) was prepared in a 60 mL polypropylene container. Simultaneously, a solution of CFPP (0.233 g, 0.1 mol % of PVA hydroxyl groups) was prepared in 10 mL of THF and added to the PVA solution. After mixing, a catalytic amount (3 drops) of hydrochloric acid was added, the mixture was stirred, and the resulting emulsion was kept in a thermostat at a temperature of 50 °C for 48 h. The gel formed after that was washed several times with distilled water to remove THF and hydrochloric acid.

### 2.6. Preparation of Silver-Containing Gel Based on PVA and CFPP

The gel obtained in Section 2.5 was loaded into a 250 mL beaker and treated with an aqueous solution of potassium hydroxide (0.48 g of KOH in 30 mL of distilled water) with stirring at a temperature of 25 °C for 48 h. The unreacted KOH was removed by washing the gel more than 3 times with distilled water. Then, the gel was treated with a previously prepared aqueous solution of silver nitrate (0.12 g of silver nitrate in 10 mL of distilled water) with stirring at a temperature of 25 °C for 48 h. The gel was washed with distilled water to remove excess silver nitrate and freeze-dried.

### 2.7. In Vivo Study: Wound-Healing Activity

The study was performed using Pannon rabbits, with characteristic clinical cases of skin and soft tissue injuries, aged from 2 weeks to 1 year (number of rabbits = 12), with four in each group. The housing and feeding conditions were standard. The injury wounds were washed with 3% hydrogen peroxide, and then with saline. The adult animals were administered with the antibiotic Nitox, at a dose of 0.2 mg/kg, and probiotics. The rabbits received only the probiotics up to 1.5 months of age. The test compounds were fixed with absorbable suture material, and sterile dressings were applied.

Type 1 treatment:

A freeze-dried aerogel in the form of a crushed sponge was placed into the wound.

Type 2 treatment:

Collagen shavings (collagen type I and II, 50:50 wt. %), prehydrated in saline for 2–3 min, were placed into the wound. A freeze-dried aerogel in the form of a crushed sponge was applied over the collagen shavings in a ratio of 90:10 wt. %, respectively.

## 3. Results and Discussion

### 3.1. Synthesis of Phosphazene-Containing Cross-Linking Agent

**Figure 1:**
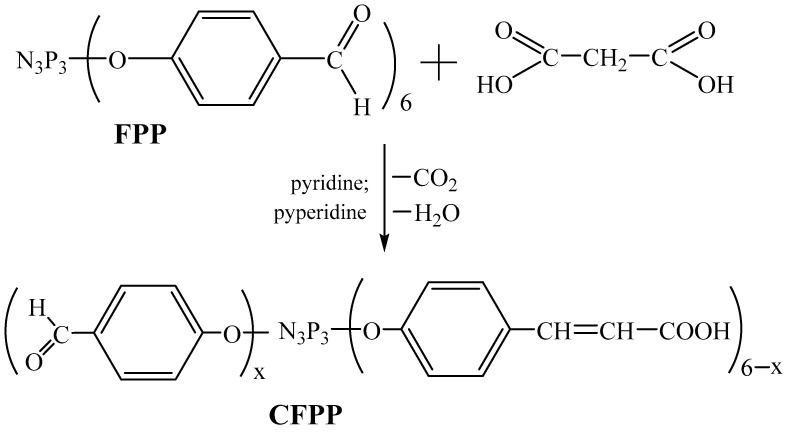
Scheme of synthesis of CFPP (x = 1–5).

Aryloxyclophosphazene CFPP containing *β*-carboxyethenylphenoxy and *p*-formylphenoxy groups was synthesized through the Doebner reaction, which is the condensation of FPP with a compound containing an active methylene group, malonic acid (MA), in the presence of a base (Figure 1).

Anhydrous pyridine was chosen as the reaction medium, since both the reactants and the final product are readily soluble in pyridine. Piperidine, which has a fairly high basicity, was used as a catalyst. The intention was to synthesize three phosphazene-containing formyl groups and three *β*-carboxyethenylphenoxy groups; hence, the initial FPP:MA molar ratio was 1:3.

**Figure 2:**
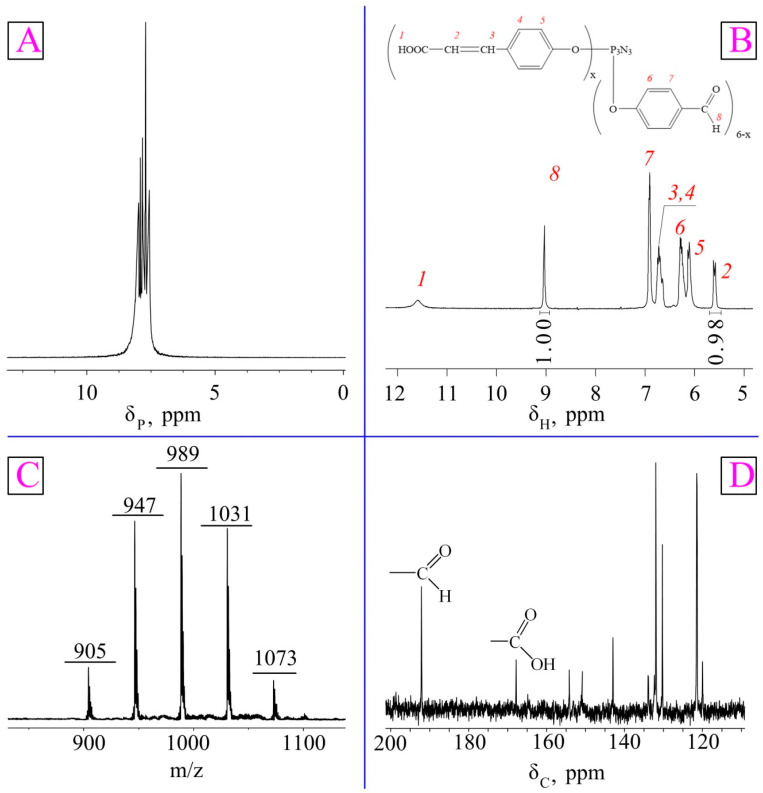
^31^P (**A**), ^1^H (**B**), and ^13^C (**D**) NMR and MALDI-TOF (**C**) spectra CFPP.

The structure of the obtained phosphazene was confirmed using phosphorus, proton, and carbon NMR spectroscopy. The phosphorus NMR spectrum of phosphazene (Figure 2A) shows a multiplet in the region of 7.56–8.27 ppm instead of a singlet, which indicates long-range interactions between the phosphorus atoms in the phosphazene ring. It may also indirectly indicate the formation of mixed reaction products, which is statistically quite likely.

Since it was difficult to detect the carboxyl groups in the ^1^H NMR spectrum due to deuterium exchange (Figure 2B), carbon NMR spectroscopy was performed, which confirmed the partial conversion of aldehyde groups to β-carboxyethenyl groups (Figure 2D).

Therefore, the ratio of the integral intensities of the proton signals of the aldehyde group and the β-carboxyethenyl double bond (Figure 2B) indicates that the contents of these groups in the product were approximately equal.

Meanwhile, it follows from the MALDI-TOF spectrum (Figure 2C) that the product was a mixture of derivatives containing one (905 *m*/*z*), two (947 *m*/*z*), three (989 *m*/*z*), four (1031 *m*/*z*), or five (1073 *m*/*z*) β-carboxyethenyl groups per phosphazene molecule. However, each of these molecules can both bind to PVA and be a silver carrier.

### 3.2. Preparation of a Silver-Containing Gel

From the literature [54], it is known that for the manifestation of an antimicrobial effect, it is necessary that the silver content in wound dressings be 0.25 wt. % relative to the weight of the polymer. Therefore, to obtain a silver-containing gel, 0.1 mol. % CFPP was used with respect to the hydroxyl groups of PVA. On the basis of this ratio, the total content of CFPP-bound silver in the gel should reach about 1.4 wt. %. This content should provide a high antimicrobial effect.

**Figure 3:**
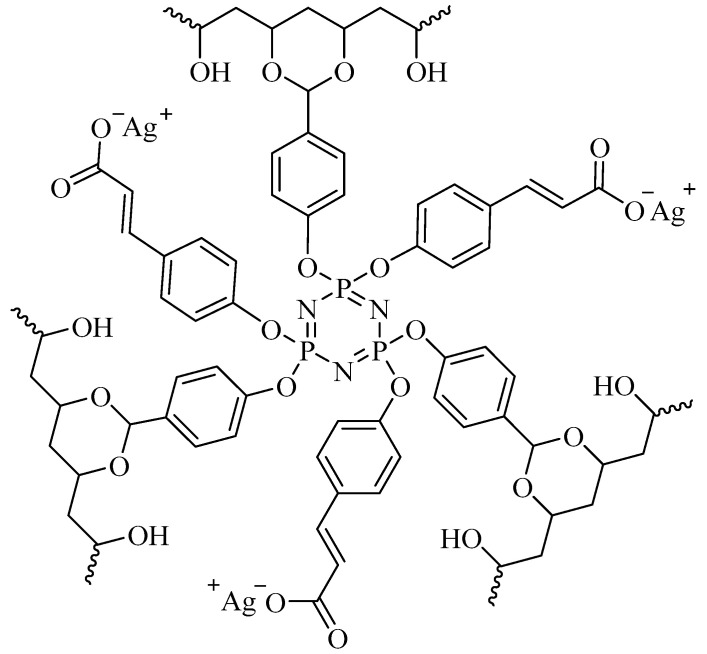
Structure of silver-containing gel.

The condensation of CFPP with PVA was carried out by mixing their solutions in THF and water, respectively. Due to the miscibility of the solvents, a stable emulsion was formed, which ensured a uniform distribution of the components in the bulk and the formation of a gel homogeneous in structure and composition. Next, the gel was thoroughly washed to remove low-molecular-weight substances and sequentially treated with alkali and silver nitrate. As a result, silver was evenly distributed throughout the polymer matrix and was chemically fixed in it in the form of cinnamates. A conditional fragment of the structure of the obtained silver-containing gel is shown in Figure 3.

The product was thoroughly washed with water, frozen, and freeze-dried to avoid coalescence.

**Figure 4:**
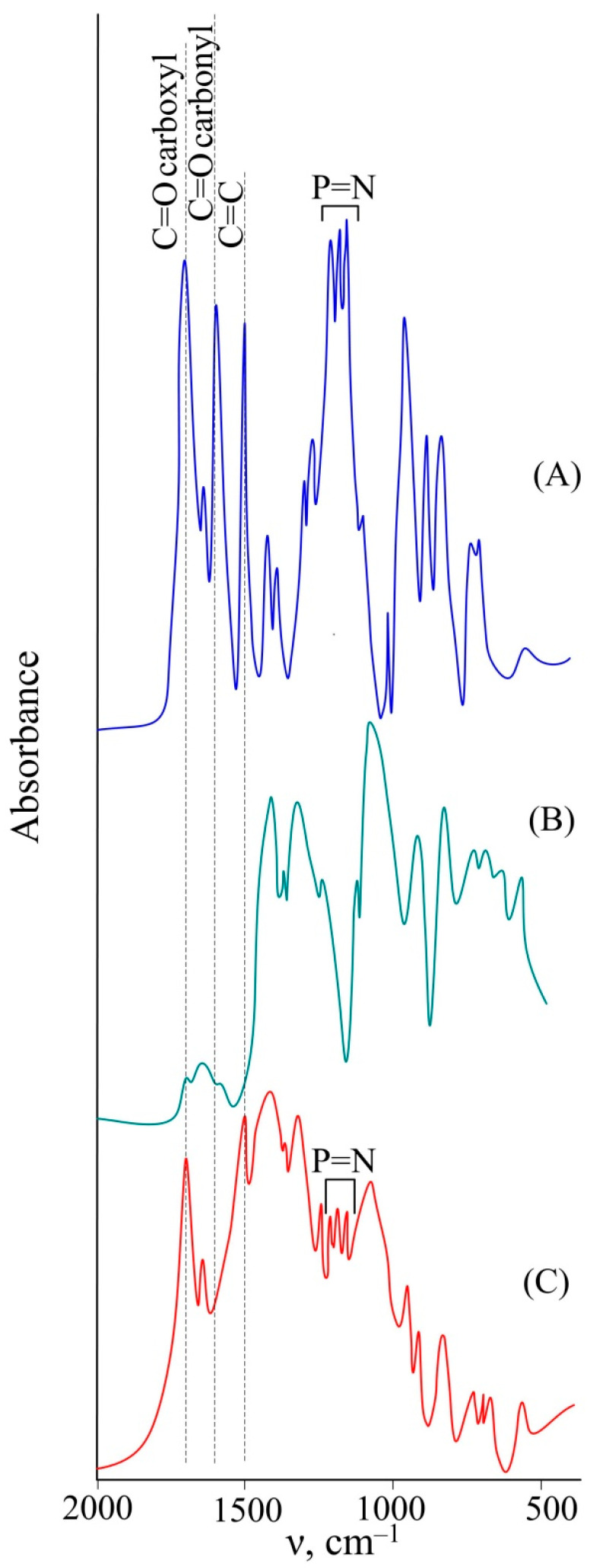
IR spectra of CFPP (**A**), PVA (**B**), and synthesized gel (**C**).

To confirm the structure of the gel, IR spectroscopy was used (Figure 4), with the initial spectra of CFPP and PVA being compared with the spectrum of the final product. It follows from the analysis of the spectra that during gel formation, the CFPP carbonyl groups reacted with the PVA macromolecules. This is confirmed by the absence of the C=O stretching modes of the aldehyde groups in the spectrum of the product. Meanwhile, the C=O stretching vibrations of carboxyl groups and double C=C bonds of CFPP can also be observed in the spectrum. Also, in the process of gel formation, the phosphazene ring was preserved, as evidenced by the presence of characteristic -P=N vibration bands in the spectrum of the gel.

The formation of a three-dimensional cross-linked gel structure was confirmed by successive extraction of soluble substances from the material with water and THF. In both cases, the content of the soluble fraction did not exceed 0.2 wt. % (within the experimental error).

The fact that phosphazene was completely incorporated into the gel during the synthesis and washing was confirmed using energy-dispersive X-ray spectroscopy (EDS). Considering the elemental composition, namely the ratio of carbon to phosphorus in the gel, and the similarity of the actual and theoretical contents of these elements, one can conclude that the initial phosphazene was completely incorporated into the gel (Table 1).

By means of EDS, it was determined that the silver content in the gel was 0.87 wt. %, which is lower than the calculated value. Nevertheless, this silver content is quite sufficient for antimicrobial behavior [54].

**Figure 5:**
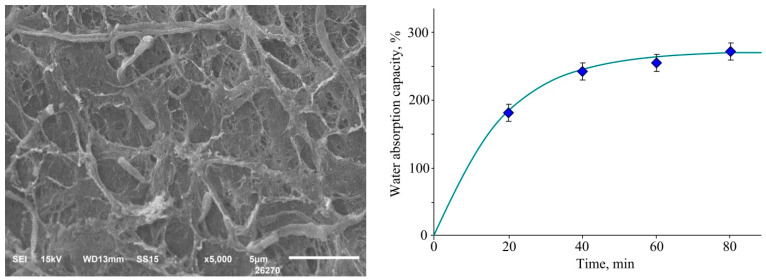
Micrograph of a freeze-dried silver gel (**left**) and its water absorption versus time (**right**).

The SEM examination of the structure of the gel revealed that the gel had open pores and was, in fact, a sponge that could absorb liquids, such as the exudate (Figure 5, left).

To confirm the possibility of sorption of liquids, the water absorption of the gel was evaluated. It was established that the water absorption capacity reached a maximum value of 272 wt. % within 80 min (Figure 5, right). In this case, the density of the aerogel sample was 0.15 g/cm^3^.

### 3.3. Study of the Antimicrobial Activity of the Silver-Containing Gel

The main opportunistic microorganisms that are most often in contact with the skin are *Escherichia coli*, *Staphylococcus aureus*, *Pseudomonas aeruginosa*, *Candida albicans*, *Bacillus subtilis*, *Staphylococcus epidermidis*, and *Corynebacterium stationis*; hence, the antimicrobial activity was evaluated against these strains.

**Figure 6:**
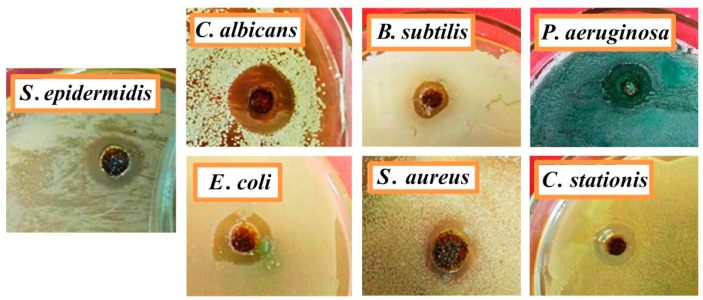
Image of zones of growth inhibition of microorganisms by a silver-containing gel on a nutrient medium.

The silver-free gel sample showed no antimicrobial activity against any of the test microorganisms. On the contrary, the silver-containing gel sample possessed antimicrobial activity against all test microorganisms with clearly defined zones of inhibition (Figure 6). Table 2 shows that the highest antimicrobial activity was observed against the Gram-negative bacteria *P. aeruginosa* and yeast-like fungus *C. albicans*. The most resistant test organisms were *B. subtilis* and *S. aureus.*

The antimicrobial activity of gel samples is associated with differences in the outer membrane and the chemical nature of pathogens. For example, the higher antimicrobial activity against Gram-negative bacteria *P. aeruginosa* compared to Gram-positive bacteria *S. aureus*, which are the main pathogens in a wound, is due to the fact that Gram-negative bacteria have a thinner peptidoglycan layer of the cell wall. This makes them more vulnerable to silver ions than Gram-positive bacteria. In addition, Gram-positive bacteria tend to form large colonies, which increases their protective properties.

### 3.4. Wound Healing Study

The wound-healing ability of the developed gel was investigated in relation to Pannon rabbits. Initially, a freeze-dried aerogel (treatment type 1) was used in the form of a sponge, which was previously crushed with a sterile tool. The gel was completely saturated with tissue fluids and blood while maintaining its structure and stability, which ensured a tight matching to the wound shape. As a result of the treatment, it was found that the scab formed on the 5th or 6th day, which is most likely due to the overdrying of the wound, which slows down the healing process. Therefore, we decided to use a combination of the developed gel and a mixture of type I and II collagen (type 2 treatment). Collagen was pre-moistened and acted as a softening buffer between the wound and the gel.

**Figure 7:**
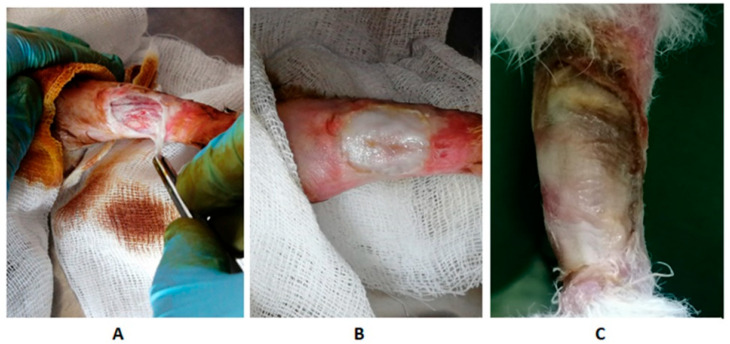
Photos of the wound during treatment using the developed gel: (**A**) damage to the left hind limb of the rabbit; (**B**) gel application (treatment type 2); (**C**) the third day after treatment.

As a result of type 2 treatment, it was revealed that on days 3–4, the wound was completely replaced by connective tissue, or formed a scab, under which the tissue was restored. No development of a secondary infection was observed at any stage of regeneration and epithelialization. The epidermal regeneration was fast in all cases and was accompanied by scar shedding (Figure 7).

No repeated operation to remove the implant material was required in either treatement. Hair growth at the site of damage started within 1–2 months.

The observations showed that, compared with the initial state, the reduction in the wound area on day 10 for the type 1 treatment group was 69.12% (*p* < 0.05), and for the type 2 treatment group, it was 91.43% (*p* < 0.05). The level of C-reactive protein in the blood serum of the animals treated according to the type 2 procedure was 1.3 times lower (*p* < 0.05) compared with those treated according to the type 1 procedure. This indicates that the most pronounced tissue regeneration and epithelialization were observed in the case of type 2 treatment.

## 4. Conclusions

The purpose of this study was to obtain a silver-containing material for wound healing that does not have a toxic effect on the human body because of the fixing of silver ions in the polymer matrix.

In conclusion, this work demonstrated that the synthesized aryloxycyclotriphosphazene containing *β*-carboxyethenylphenoxy and *p*-formylphenoxy groups is a promising compound for the preparation of a silver-containing gel, since it acts both as a cross-linking agent and as an agent for binding silver ions, contributing to their uniform distribution throughout the polymer matrix without aggregation or side effects. The resulting silver-containing aerogel based on polyvinyl alcohol and phosphazene has good porosity, a high water absorption (272 wt. % in 80 min), and antimicrobial and wound-healing effects. An appropriate porosity and degree of swelling would generate a moist wound environment and impart the ability to absorb and retain the wound exudate. The aerogel containing 0.87 wt. % silver showed inhibitory activity against the tested pathogens that infect the wound (the bacteria *S. aureus*, *P. aeruginosa*, *E. coli*, *B. subtilis*, *S. epidermidis*, and *C. stationis* and the fungus *C. albicans*). An *in vivo* study of the aerogel using Pannon rabbits (n = 12) demonstrated an excellent wound-healing effect consisting of rapid regeneration of the epidermis as early as 3–4 days after the treatment was applied. The resulting aerogel is recommended for use as an innovative wound dressing for healing acute and open wounds.

## Figures and Tables

**Table 1:** The content of elements in dry gel in wt. %.

Element	Actual Content	Theoretical Content
C	55.74	54.70
P	0.41	0.40
Ag	0.87	1.41

**Table 2:** Antimicrobial action of silver-containing gel.

Microorganisms	Inhibition Zone Diameter, mm
*E. coli*	13–14
*S. aureus*	12–13
*P. aeruginosa*	19–20
*C. albicans*	20–21
*S. epidermidis*	14–15
*C. stationis*	13–14
*B. subtilis*	9–10

## Data Availability

Not applicable.

## References

[1] Burgess J.L., Wyant W.A., Abdo Abujamra B., Kirsner R.S., Jozic I. (2021). Diabetic Wound-Healing Science. Medicina.

[2] Gowda B.H.J., Mohanto S., Singh A., Bhunia A., Abdelgawad M.A., Ghosh S., Ansari M.J., Pramanik S. (2023). Nanoparticle-based therapeutic approaches for wound healing: A review of the state-of-the-art. Mater. Today Chem..

[3] Brumberg V., Astrelina T., Malivanova T., Samoilov A. (2021). Modern wound dressings: Hydrogel dressings. Biomedicines.

[4] Xiang J., Zhu R., Lang S., Yan H., Liu G., Peng B. (2021). Mussel-inspired immobilization of zwitterionic silver nanoparticles toward antibacterial cotton gauze for promoting wound healing. Chem. Eng. J..

[5] Demeter M., Scărișoreanu A., Călina I. (2023). State of the Art of Hydrogel Wound Dressings Developed by Ionizing Radiation. Gels.

[6] Alfuraydi R.T., Alminderej F.M., Mohamed N.A. (2022). Evaluation of Antimicrobial and Anti-Biofilm Formation Activities of Novel Poly(vinyl alcohol) Hydrogels Reinforced with Crosslinked Chitosan and Silver Nano-Particles. Polymers.

[7] Semenova M.V., Osadchenko S.V., Mezhuev Y.O., Shtil’man M.I., Semenova I.N. (2016). Synthesis of hemocompatible materials based on branched polyvinyl alcohol. Russ. J. Appl. Chem..

[8] Mezhuev Y.O., Varankin A.V., Luss A.L., Dyatlov V.A., Tsatsakis A.M., Shtilman M.I., Korshak Y.V. (2020). Immobilization of dopamine on the copolymer of N-vinyl-2-pyrrolidone and allyl glycidyl ether and synthesis of new hydrogels. Polym. Int..

[9] Shen Z., Zhang C., Wang T., Xu J. (2023). Advances in Functional Hydrogel Wound Dressings: A Review. Polymers.

[10] Alamoudi A.A., Alharbi A.S., Abdel-Naim A.B., Badr-Eldin S.M., Awan Z.A., Okbazghi S.Z., Ahmed O.A.A., Alhakamy N.A., Fahmy U.A., Esmat A. (2022). Novel Nanoconjugate of Apamin and Ceftriaxone for Management of Diabetic Wounds. Life.

[11] Liu W.S., Liu Y., Gao J., Zheng H., Lu Z.M., Li M. (2023). Biomembrane-Based Nanostructure-and Microstructure-Loaded Hydrogels for Promoting Chronic Wound Healing. Int. J. Nanomed..

[12] Sari M.H.M., Cobre A.D.F., Pontarolo R., Ferreira L.M. (2023). Status and Future Scope of Soft Nanoparticles-Based Hydrogel in Wound Healing. Pharmaceutics.

[13] Sabbagh F., Kim B.S. (2022). Recent advances in polymeric transdermal drug delivery systems. J. Control. Release.

[14] Morozova S.M. (2023). Recent Advances in Hydrogels via Diels–Alder Crosslinking: Design and Applications. Gels.

[15] Gherman S.P., Biliuță G., Bele A., Ipate A.M., Baron R.I., Ochiuz L., Șpac A.F., Zavastin D.E. (2023). Biomaterials Based on Chitosan and Polyvinyl Alcohol as a Drug Delivery System with Wound-Healing Effects. Gels.

[16] Feraru A., Tóth Z.-R., Mureșan-Pop M., Baia M., Gyulavári T., Páll E., Turcu R.V.F., Magyari K., Baia L. (2023). Anionic Polysaccharide Cryogels: Interaction and In Vitro Behavior of Alginate–Gum Arabic Composites. Polymers.

[17] Mary S.A., Ariram N., Gopinath A., Chinnaiyan S.K., Raja I.S., Sahu B., Giri Dev V.R., Han D.-W., Madhan B. (2023). Investigation on Centrifugally Spun Fibrous PCL/3-Methyl Mannoside Mats for Wound Healing Application. Polymers.

[18] Janů L., Dvořáková E., Polášková K., Buchtelová M., Ryšánek P., Chlup Z., Kruml T., Galmiz O., Nečas D., Zajíčková L. (2023). Enhanced Adhesion of Electrospun Polycaprolactone Nanofibers to Plasma-Modified Polypropylene Fabric. Polymers.

[19] Ayvazoğlu B.Ş., Ceylan M., Turan A.A.I., Yılmaz E.B. (2023). Biodegradable Polycaprolactone Fibers with Silica Aerogel and Nanosilver Particles Produce a Coagulation Effect. Polymers.

[20] Liu Y., Li C., Feng Z., Han B., Yu D.G., Wang K. (2022). Advances in the Preparation of Nanofiber Dressings by Electrospinning for Promoting Diabetic Wound Healing. Biomolecules.

[21] Allizond V., Banche G., Salvoni M., Malandrino M., Cecone C., Cuffini A.M., Bracco P. (2023). Facile One-Step Electrospinning Process to Prepare AgNPs-Loaded PLA and PLA/PEO Mats with Antibacterial Activity. Polymers.

[22] Pessanha F.S., Oliveira B.G.R.B.d., Oliveira B.C., Deutsch G., Teixeira F.L., Bokehi L.C., Calomino M.A., Rodrigues de Castilho S., Thiré R.M.D.S.M., Teixeira L.A. (2023). Effectiveness of Epidermal Growth Factor Loaded Carboxymethylcellulose (EGF-CMC) Hydrogel in Biofilm Formation in Wounds of Diabetic Patients: A Randomized Clinical Trial. Gels.

[23] Cai K., Liu Y., Yue Y., Liu Y., Guo F. (2023). Essential Oil Nanoemulsion Hydrogel with Anti-Biofilm Activity for the Treatment of Infected Wounds. Polymers.

[24] Suneetha M., Won S.-Y., Zo S.M., Han S.S. (2023). Fungal Carboxymethyl Chitosan-Impregnated Bacterial Cellulose Hydrogel as Wound-Dressing Agent. Gels.

[25] Rao K.M., Uthappa U.T., Kim H.J., Han S.S. (2023). Tissue Adhesive, Biocompatible, Antioxidant, and Antibacterial Hydrogels Based on Tannic Acid and Fungal-Derived Carboxymethyl Chitosan for Wound-Dressing Applications. Gels.

[26] Stanescu P.O., Radu I.C., Leu Alexa R., Hudita A., Tanasa E., Ghitman J., Stoian O., Tsatsakis A., Ginghina O., Zaharia C. (2021). Novel chitosan and bacterial cellulose biocomposites tailored with polymeric nanoparticles for modern wound dressing development. Drug Deliv..

[27] Afzal A., Jalalah M., Noor A., Khaliq Z., Qadir M.B., Masood R., Nazir A., Ahmad S., Ahmad F., Irfan M. (2023). Development and Characterization of Drug Loaded PVA/PCL Fibres for Wound Dressing Applications. Polymers.

[28] Capanema N.S.V., Mansur A.A.P., Carvalho I.C., Carvalho S.M., Mansur H.S. (2023). Bioengineered Water-Responsive Carboxymethyl Cellulose/Poly(vinyl alcohol) Hydrogel Hybrids for Wound Dressing and Skin Tissue Engineering Applications. Gels.

[29] Valentino C., Martínez Rodríguez T., Borrego-Sánchez A., Hernández Benavides P., Arrebola Vargas F., Paredes J.M., Rossi S., Sainz Díaz C.I., Sandri G., Grisoli P. (2023). Characterization and Molecular Modelling of Non-Antibiotic Nanohybrids for Wound Healing Purposes. Pharmaceutics.

[30] Cooper M.L., Laxer J.A., Hansbrough J.F. (1991). The cytotoxic effects of commonly used topical antibacterial agents on human fibroblasts and keratinocytes. J. Trauma.

[31] Elbagory A.M., Meyer M., Cupido C.N., Hussein A.A. (2017). Inhibition of Bacteria Associated with Wound Infection by Biocompatible Green Synthesized Gold Nanoparticles from South African Plant Extracts. Nanomaterials.

[32] Ferrara F., Benedusi M., Cervellati F., Sguizzato M., Montesi L., Bondi A., Drechsler M., Pula W., Valacchi G., Esposito E. (2022). Dimethyl Fumarate-Loaded Transethosomes: A Formulative Study and Preliminary Ex Vivo and In Vivo Evaluation. Int. J. Mol. Sci..

[33] Ceylan S., Küçükosman R., Yurt F., Özel D., Öztürk İ., Demir D., Ocakoglu K. (2022). Antimicrobial activity enhancement of PVA/chitosan films with the additive of CZTS quantum dots. Polym. Bull..

[34] Phetcharat P., Sangsanoh P., Choipang C., Chaiarwut S., Suwantong O., Chuysinuan P., Supaphol P. (2023). Curative Effects of Copper Iodide Embedded on Gallic Acid Incorporated in a Poly (Vinyl Alcohol)(PVA) Liquid Bandage. Gels.

[35] Mallick S., Nag M., Lahiri D., Pandit S., Sarkar T., Pati S., Nirmal N.P., Edinur H.A., Kari Z.A., Ahmad Mohd Zain M.R. (2022). Engineered Nanotechnology: An Effective Therapeutic Platform for the Chronic Cutaneous Wound. Nanomaterials.

[36] Melnikova N., Knyazev A., Nikolskiy V., Peretyagin P., Belyaeva K., Nazarova N., Liyaskina E., Malygina D., Revin V. (2021). Wound Healing Composite Materials of Bacterial Cellulose and Zinc Oxide Nanoparticles with Immobilized Betulin Diphosphate. Nanomaterials.

[37] Balcucho J., Narváez D.M., Tarazona N.A., Castro-Mayorga J.L. (2023). Microbially Synthesized Polymer-Metal Nanoparticles Composites as Promising Wound Dressings to Overcome Methicillin-Resistance *Staphylococcus aureus* Infections. Polymers.

[38] Almaieli L.M.A., Khalaf M.M., Gouda M., Elmushyakhi A., Abou Taleb M.F., El-Lateef A., Hany M. (2023). Fabrication of Bio-Based Film Comprising Metal Oxide Nanoparticles Loaded Chitosan for Wound Dressing Applications. Polymers.

[39] Sabarees G., Velmurugan V., Tamilarasi G.P., Alagarsamy V., Raja Solomon V. (2022). Recent Advances in Silver Nanoparticles Containing Nanofibers for Chronic Wound Management. Polymers.

[40] Xiang Y., Qi X., Cai E., Zhang C., Wang J., Lan Y., Deng H., Shen J., Hu R. (2023). Highly efficient bacteria-infected diabetic wound healing employing a melanin-reinforced biopolymer hydrogel. Chem. Eng. J..

[41] Pino P., Bosco F., Mollea C., Onida B. (2023). Antimicrobial Nano-Zinc Oxide Biocomposites for Wound Healing Applications: A Review. Pharmaceutics.

[42] Sajjad A., Sajjad H., Hanif S., Rasheed F., Zia M. (2023). Fabrication and characterization of wheat-gluten/hematite nanocomposite film with antibacterial and antioxidant properties for biological applications. Biomass Convers. Biorefin..

[43] Díaz-Puertas R., Rodríguez-Cañas E., Bello-Perez M., Fernández-Oliver M., Mallavia R., Falco A. (2023). Viricidal Activity of Thermoplastic Polyurethane Materials with Silver Nanoparticles. Nanomaterials.

[44] Júnior D.M., Hausen M.A., Asami J., Higa A.M., Leite F.L., Mambrini G.P., Rossi A.L., Komatsu D., Duek E.A.D.R. (2021). A New Dermal Substitute Containing Polyvinyl Alcohol with Silver Nanoparticles and Collagen with Hyaluronic Acid: In Vitro and In Vivo Approaches. Antibiotics.

[45] Nosrati H., Heydari M., Tootiaei Z., Ganjbar S., Khodaei M. (2023). Delivery of antibacterial agents for wound healing applications using polysaccharide-based scaffolds. J. Drug Deliv. Sci. Technol..

[46] Mehrabi T., Mesgar A.S., Mohammadi Z. (2020). Bioactive glasses: A promising therapeutic ion release strategy for enhancing wound healing. ACS Biomater. Sci. Eng..

[47] Velnar T., Bailey T., Smrkolj V. (2009). The wound healing process: An overview of the cellular and molecular mechanisms. J. Int. Med. Res..

[48] Zhu J., Zhou H., Gerhard E.M., Zhang S., Rodríguez F.I.P., Pan T., Cheng H. (2023). Smart bioadhesives for wound healing and closure. Bioact. Mater..

[49] Chiangnoon R., Karawak P., Eamsiri J., Nuchdang S., Thamrongsiripak N., Neramitmansook N., Pummarin S., Pimton P., Nilgumhang K., Uttayarat P. (2023). Antibacterial Hydrogel Sheet Dressings Composed of Poly(vinyl alcohol) and Silver Nanoparticles by Electron Beam Irradiation. Gels.

[50] Massironi A., Franco A.R., Babo P.S., Puppi D., Chiellini F., Reis R.L., Gomes M.E. (2022). Development and Characterization of Highly Stable Silver NanoParticles as Novel Potential Antimicrobial Agents for Wound Healing Hydrogels. Int. J. Mol. Sci..

[51] Li R., Qi Q., Wang C., Hou G., Li C. (2023). Self-Healing Hydrogels Fabricated by Introducing Antibacterial Long-Chain Alkyl Quaternary Ammonium Salt into Marine-Derived Polysaccharides for Wound Healing. Polymers.

[52] Khansa I., Schoenbrunner A.R., Kraft C.T., Janis J.E. (2019). Silver in wound care—Friend or foe?: A comprehensive review. Plast. Reconstr. Surg. Glob. Open.

[53] Chistyakov E.M., Kireev V.V., Filatov S.N., Terekhov I.V., Buzin M.I., Komarova L.I. (2012). Thermal polycondensation of hexa-p-hydroxymethylphenoxycyclotriphosphazene. Polym. Sci. Ser. B.

[54] Oliveira R.N., Rouzé R., Quilty B., Alves G.G., Soares G.D.A., Thiré R.M.S.M., McGuinness G.B. (2014). Mechanical properties and in vitro characterization of polyvinyl alcohol-nano-silver hydrogel wound dressings. Interface Focus.

